# Toward uncharted territory of cellular heterogeneity: advances and applications of single-cell RNA-seq

**DOI:** 10.20517/jtgg.2020.51

**Published:** 2021-01-01

**Authors:** Brandon Lieberman, Meena Kusi, Chia-Nung Hung, Chih-Wei Chou, Ning He, Yen-Yi Ho, Josephine A. Taverna, Tim H. M. Huang, Chun-Liang Chen

**Affiliations:** 1 Department of Molecular Medicine, University of Texas Health Science Center at San Antonio, San Antonio, TX 78229, USA.; 2 Department of Nursing, University of Texas Health Science Center at San Antonio, San Antonio, TX 78229, USA.; 3 Department of Statistics, University of South Carolina, Columbia, SC 29208, USA.; 4 Department of Medicine, University of Texas Health Science Center at San Antonio, San Antonio, TX 78229, USA.; 5 Mays Cancer Center, University of Texas Health Science Center at San Antonio, San Antonio, TX 78229, USA.

**Keywords:** Single-cell RNA-seq, transcriptome, heterogeneity, multiplexing, high throughput, dimensional reduction, cancer, diseases

## Abstract

Among single-cell analysis technologies, single-cell RNA-seq (scRNA-seq) has been one of the front runners in technical inventions. Since its induction, scRNA-seq has been well received and undergone many fast-paced technical improvements in cDNA synthesis and amplification, processing and alignment of next generation sequencing reads, differentially expressed gene calling, cell clustering, subpopulation identification, and developmental trajectory prediction. scRNA-seq has been exponentially applied to study global transcriptional profiles in all cell types in humans and animal models, healthy or with diseases, including cancer. Accumulative novel subtypes and rare subpopulations have been discovered as potential underlying mechanisms of stochasticity, differentiation, proliferation, tumorigenesis, and aging. scRNA-seq has gradually revealed the uncharted territory of cellular heterogeneity in transcriptomes and developed novel therapeutic approaches for biomedical applications. This review of the advancement of scRNA-seq methods provides an exploratory guide of the quickly evolving technical landscape and insights of focused features and strengths in each prominent area of progress.

## INTRODUCTION

**Figure 1.**
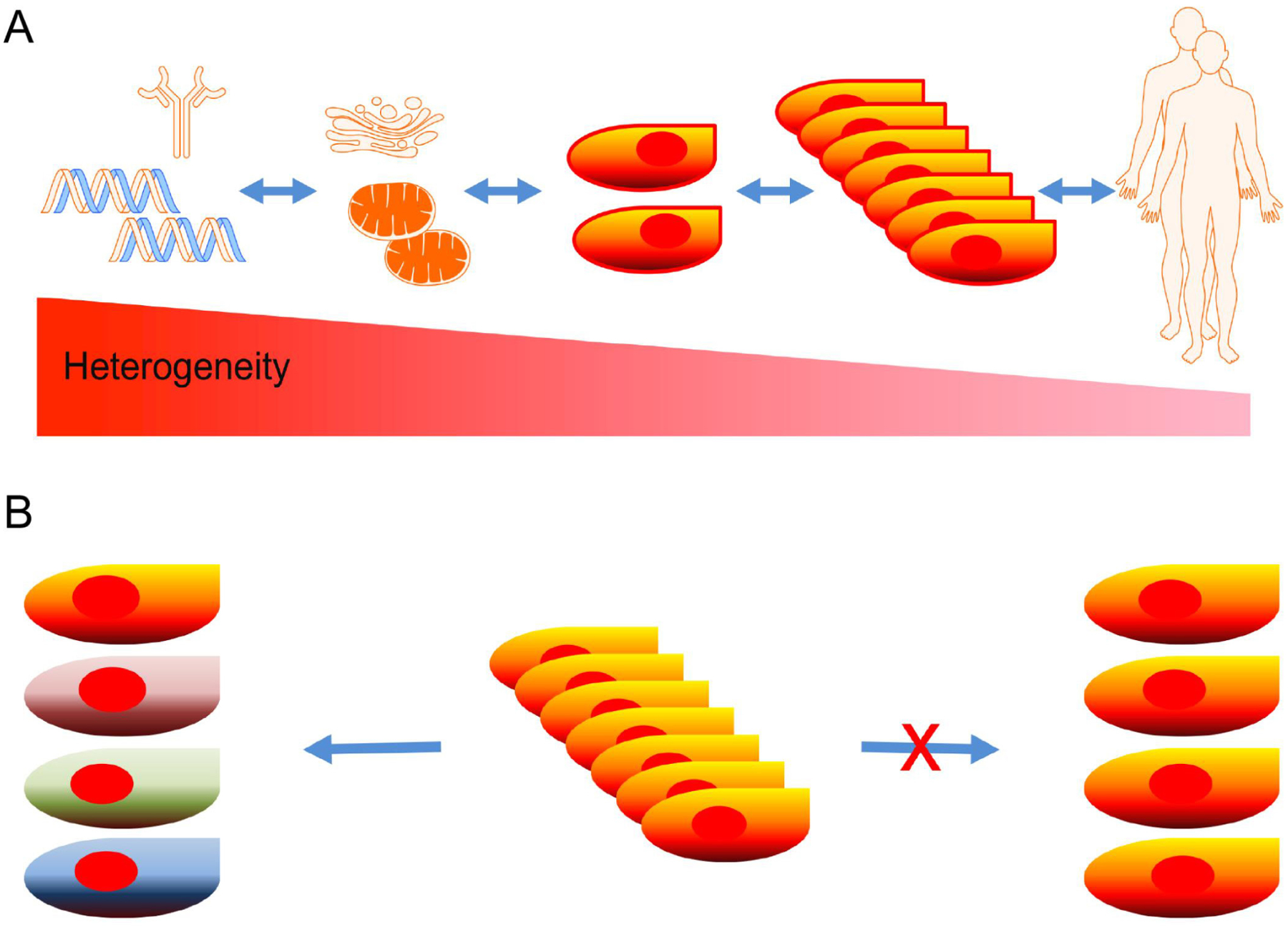
A new paradigm for cellular heterogeneity: heterogeneity and homology coexistent in all levels of phenotypes and genotypes in humans, as heterogeneity is increased from individual level down to molecular level (A); a new paradigm predicts that cells from the same tissue are not created equally and heterogeneity of cells are far more than we previously perceived based on bulk studies (B)

Homogeneity and heterogeneity are proportionally co-existent in all phenotypical and genetic levels of humans. The extent of heterogeneity is increased from individuals down to molecules, whereas homogeneity is decreased [Figure 1A]. The diversity is observed in individuals, organs, tissues, cells, organelles, and molecules, and even more abundant in protein, DNA, and RNA molecules. The long-standing paradigm that cells of the same tissue origin are homogeneous based on bulk cell studies has lately been challenged by single-cell studies^[1–5]^. New data show that cells arising from the same tissue origins are far more heterogeneous than they seemingly appear [Figure 1B]^[6–8]^. Even genetically identical cells cultured in the same conditions have shown variations in gene expression^[9,10]^.

In the new paradigm, the diverse properties of cells are mainly reflected in the heterogeneous gene expression, genomic alterations, epigenomic modifications, and proteomic fluctuations^[7,11–16]^. Cellular transcriptomic heterogeneity helped to establish a new paradigm of cellular heterogeneity with the invention of scRNA-seq^[17–19]^. The cellular transcriptomic heterogeneity arises from stochasticity, differentiation, environmental stimuli, diseases, aging, and other factors^[5,8,11,20–23]^. The development of single-cell analysis was overshadowed by traditional bulk cell approaches and technically limited by the absence of high throughput single-cell isolation and minute initiation materials (picogram DNA and mRNA per cell)^[3]^. Combined technological advances in cell isolation, high throughput multiplexing, amplification, and next generation sequencing facilitated scRNA-seq and uncovered cellular heterogeneity^[24]^. Mapping transcriptomic changes at single-cell level has since revealed global gene expression profiles and exposed stochasticity, differentiation, cell fate plasticity, and diseases^[25]^. In this review, we highlight the novel scRNA-seq platforms and conduct a comparative analysis of each technology and their future applications in translational science.

## SINGLE-CELL ISOLATION TECHNOLOGIES

Cell purity is paramount for scRNA-seq and other single-cell analysis methods. Tissues, organoids, and 2D and 3D cultured cells are multi-cellular, and the first step to dissociate aggregated cells into individual cells risks potential contamination with cell doublets, DNA, and RNA by incomplete enzymatic digestion and cell lysis. The impurity of single cells distorts the scRNA-seq data and leads to false interpretations. To ensure the purity and integrity of single cells, several instrumental technologies have been adopted to overcome these technical challenges.

### Manual cell picking (micromanipulation)

Manual cell picking presents a simple and cost-effective method for single-cell isolation. This technique involves an inverted fluorescent microscope, manipulator, and microinjector for precise cell location and picking after cells are labeled with markers^[26]^. The instrumental efficiency of picking individual cells exceeds old-fashioned mouth-pipetting^[17]^. Specifically, cells are maintained in suspension and manually isolated by capillary pipettes connected with a microinjector. Cellular integrity is maintained for further analysis and is particularly workable with rare cells. However, high operator skills are required through training and practice. Additionally, the throughput is relatively low compared with the other methods^[27]^.

### Flow activated cell sorting

This high throughput method relies on antibody affinity to cell surface markers and has become the most common strategy for single-cell isolation. Cells are labeled with fluorescent or conjugated antibodies and run through flow cytometry, sensed by laser detectors or a magnetic field, and sorted with surface specific markers^[27,28]^. With advanced fluorochrome and microscope techniques, 18 fluorescent, inorganic semiconductor nanocrystals (Quantum Dots) are used to label antigens on cells, which increases the specificity and sensitivity of single-cell isolation from a bulk sample^[29,30]^. However, greater than 10,000 cells are required for this method and signal overlap may affect the purity of the target cells. Moreover, this method cannot perform single-cell analysis with rare cells.

### Microfluidic technology

This technology for single-cell isolation can be divided into three main approaches: droplet-based microfluidics, channel-based microfluidics, and hydrodynamic traps. These methods rely on cell adhesion, hydrodynamics, physical characteristics (e.g., size and shape), cellular density, and elasticity. Microfluidic technology platforms can actively or passively recognize and sort single cells from a heterogenic population^[31]^. In droplet-based microfluidics, each single cell is embedded in a hydrophilic droplet which suspends hydrophobic channels. The advantages of this approach are high throughput and yield, making it feasible to isolate rare cell types^[32]^. Besides, genetic barcodes can be added within the cell droplet that record the cell origin, allowing profiling of cells from simultaneous preparation of thousands of single-cell libraries^[33,34]^. In channel-based microfluidics, single cell is controlled and confined by pneumatic membrane valves according to the biological requirements. This selection approach increases the accuracy of cell isolation and the flexibility of experimental design. However, it is limited by the low throughput compared with droplet-based microfluidics. Hydrodynamic traps such as Fluidigm C1 passively isolate and trap single cells based on cell size^[31,32]^. Both channel-based microfluidics and hydrodynamic traps enable long-term cell culture and high-resolution observation for further biological experiments such as drug treatment or cDNA library preparation^[35]^.

### Laser capture microdissection

This microscopy-based technology carries out isolation of specific single cells on a microscope slide without cell dissociation from solid samples. Tissue sections are either top-covered by or laid on a thermoplastic polymer film, which is heat-activated by infrared or ultraviolet laser. The boundaries of a single cell on a tissue section are recognized and severed precisely by laser, and the dissected cell is captured^[36,37]^. Laser capture microdissection is a rapid and precise isolation technology that maintains versatility for further analysis including scRNA-seq^[38,39]^.

## TECHNICAL ADVANCES FOR CDNA SYNTHESIS AND AMPLIFICATION

The uniform and full coverage of cDNA synthesis from single-cell mRNA/RNA is a crucial step for the success of scRNA-seq because the limited starting materials are as little as 5–30 pg and need to be amplified for next generation sequencing. The cDNA synthesis from single cells has been attempted for qRT-PCR and microarrays, and the technical predecessors were adopted and modified for scRNA-seq^[40,41]^. The protocols of scRNA-seq have fruitfully advanced in a decade [Table 1]^[19,25,42–44]^. The technical variations have strengths and weaknesses in linear amplification, length coverage, low copy RNA species detection, multiplexing, high throughputs, and cost reduction. Tang’s protocol was the first scRNA-seq modality and was based on single-cell RNA amplification from RNA microarray assays^[45,46]^. Tang’s protocol uses poly(T) primers to generate full-length cDNA of transcripts less than 3 kb and can detect ~13,000 genes, 65% of microarray genes^[42]^. Two years later, Single-cell Tagged Reverse Transcription sequencing (STRT-seq) introduced template switching to incorporate bead-linked barcoded primers for strand-specific amplification of 3’ ends and high throughput 96-cell multiplexing with 2000–4000 genes detected in individual cells^[18,47,48]^. In 2012, a significant advancement for full-length cDNA synthesis of 40% transcripts was made with Smart-seq, and it was updated with the Smart-seq2 in 2013^[49–51]^. Smart-seq has laid the foundation for future scRNA-seq methods, employing more stable template switching ribo(guanosine)_3_ oligos and having more unique mapping reads, higher recovery rates of low expression genes, and a two-fold increase in spliced forms discovered. At about the same time, Cell Expression by Linear amplification and sequencing (CEL-seq) utilized linear strand-specific *in vitro* transcript amplification mainly at 3’ ends, and an improved CEL-seq2 version reduced mRNA molecule counting biases with the introduction of unique molecular identifiers (UMIs)^[52–55]^. Single Cell RNA Barcoding and sequencing (SCRB-seq) is a protocol for high throughput of 12,000 cells at a low cost and one of the first scRNA-seq protocols to include UMIs^[56]^. Previous scRNA-seq platforms utilized relative measures such as reads per kilobase per million reads (RPKM), which masked differences in total mRNA content. As an example, a gene may be “upregulated” in terms of RPKM and have a decrease in absolute expression level. UMIs are short unique sequences integrated in cDNAs before PCR amplification to allow for unique identification of amplified DNAs carrying the same UMI sharing the same mRNA/RNA molecule origin and reduce nonlinear PCR amplification bias. For full length transcript coverage and analysis of noncoding RNA, Multiple Annealing and dC-Tailing-based Quantitative single-cell RNA-seq (MATQ-seq) and Random Displacement Amplification sequencing (RamDA-seq) can be employed, which allow for poly(A)^+^ and non-poly(A) scRNA-seq, useful for characterization of lncRNA or circRNA^[57–59]^. RamDA-seq also detects enhancer RNAs differentially expressed in a cell-type specific manner. Quartz-Seq builds upon both CEL-seq2 and STRT-seq to perform 3’ coverage scRNA-seq, vastly improving poly(A) tailing and initial read UMI conversion and augmenting sequencing depth and accuracy^[60]^.

Many methods employ cDNA amplification strategies, as mentioned above, but they each have unique methods of single-cell sorting and significant high throughput improvement. MAssively parallel RNA Single-cell sequencing (MARS-seq) uses fluorescence-activated cell sorting sorting to separate and sort 100–1000 cells into individual wells^[61]^. Drop-seq and InDrop are two similar methods that employ droplet capture microfluidic methods to isolate cells. The main differences are that Drop-seq uses reagent containing beads, while InDrop uses reagent carrying hydrogel microspheres. Both platforms can quickly process tens of thousands of cells daily^[33,34]^. Chromium is similar to both previous methods, employing a gel bead in emulsion^[62]^ microfluidic capture method, but has the advantage of being able to process eight samples at once or a single sample more quickly due to its eight-channel microfluidic chip^[63]^. Seq-Well is a low-cost alternative, not requiring any expensive microfluidic devices and instead utilizing semi-permeable membranes on a picowell plate with wells that contain one barcoded capture bead and space for one single cell per well^[64]^. Seq-Well plates have ~86,000 wells, but the actual capture efficiency varies. Similar to the other platforms (CEL-seq, STRT-seq, and SCRB-seq), CytoSeq employs a microfluidic method automating high throughput cell settling in 1/10 of 100,000-well plate by gravity^[65]^. It employs a similar plate system to Seq-Well with 30-μm well sizes only allowing one cell per well and one magnetic bead with a universal primer plus 10^6^ diverse UMIs created by a split-pool synthesis process. It can easily reach up to 10,000 cells with detection of ~100 genes per cell.

For harder to work with tissues, such as frozen samples, DroNC-seq is able to salvage samples and produce high quality data, employing single nucleus RNA-seq with 3’ coverage^[66]^. sci-RNA-seq performs the analysis of single cells or nuclei isolated from methanol-fixed whole organisms (~50,000 cells), with 3’ coverage and high depth sequencing employing double UMI barcoding^[67]^. Split-Pool Ligation-based Transcriptome sequencing (SPLiT-seq) is an extremely high throughput 3’ coverage method distinct from other methods, offering scRNA-seq analysis without single-cell isolation from 1.33% formaldehyde-fixed tissues^[68]^. It employs combinatorial indexing to identify single cells without isolation, by performing three successive barcoding steps through *in situ* reverse transcription on groups of cells and mixing after each time, leaving each cell with a unique identifier totaling up to 21 million for downstream data analysis. This invention posits potential cost reduction per cell and time effectiveness.

Not all methods employ UMIs. Designed Primer-based RNA-sequencing (DP-seq) is a 3’ coverage method useful for small samples, analyzes at least 50 pg of RNA, and employs random hexamer-based amplification and sequencing^[69]^. The protocol requires knowledge of the intended target’s genome prior to use. SC3-seq provides 3’ coverage method for only 3’ end characterization of mRNA, allowing for higher reproducibility and reduced noise, thus it is useful for projects not requiring deep sequencing with a low budget^[46,70]^.

Attempts have been made to evaluate diverse scRNA-seq protocols using systematic comparisons^[71]^. Six commonly used methods - CEL-seq2, Drop-seq, MARS-seq, SCRB-seq, Smart-seq, and Smart-seq2 - were performed side by side using mouse embryonic stem cells. Overall, Smart-seq2 detected the most genes per cell, while the other methods displayed less amplification noise due to the use of UMIs. Power analysis showed that Drop-seq provides a less costly option for a larger number of cells analyzed, whereas MARS-seq, SCRB-seq, and Smart-seq2 were suitable for fewer cells. To evaluate sensitivity and accuracy as performance metrices for 15 different methods, *in silico* power analysis of External RNA Controls Consortium spike-in standards was performed in those scRNA-seq studies^[72]^. The vast majority of methods displayed high accuracy. The exceptions were CEL-seq and MARS-seq data, which show more variations among cells. Better sensitivity appeared in SMARTer (C1), CEL-Seq2 (C1), STRT-Seq, and InDrop-seq that could detect digital copies of spike-ins; however, the sensitivity was sequencing depth-dependent. For the droplet-based high-throughput platforms (InDrop, Drop-seq, and 10x Genomics Chromium), a thorough comparative study revealed insights regarding their efficacy and limits^[73]^. The 10X Genomics Chromium protocol was maturely developed with a higher cost and delivered high degree of sensitivity and accuracy with less technical noise. Drop-seq provided similar data quality with fewer cells, but a more affordable cost. InDrop is also less expensive with high compatibility with other protocols, such as Smart-seq2. In a large-scale validation of 13 protocols by multi-centered collaboration effort using mixed human and murine cells, CEL-seq2, Quartz-seq2, SMAR-seq2, and Chromium platforms were superior in producing high-resolution transcriptomic profiles^[74]^.

## PROCESSING OF NEXT GENERATION SEQUENCING DATA OF SCRNA-SEQ

**Figure 2.**
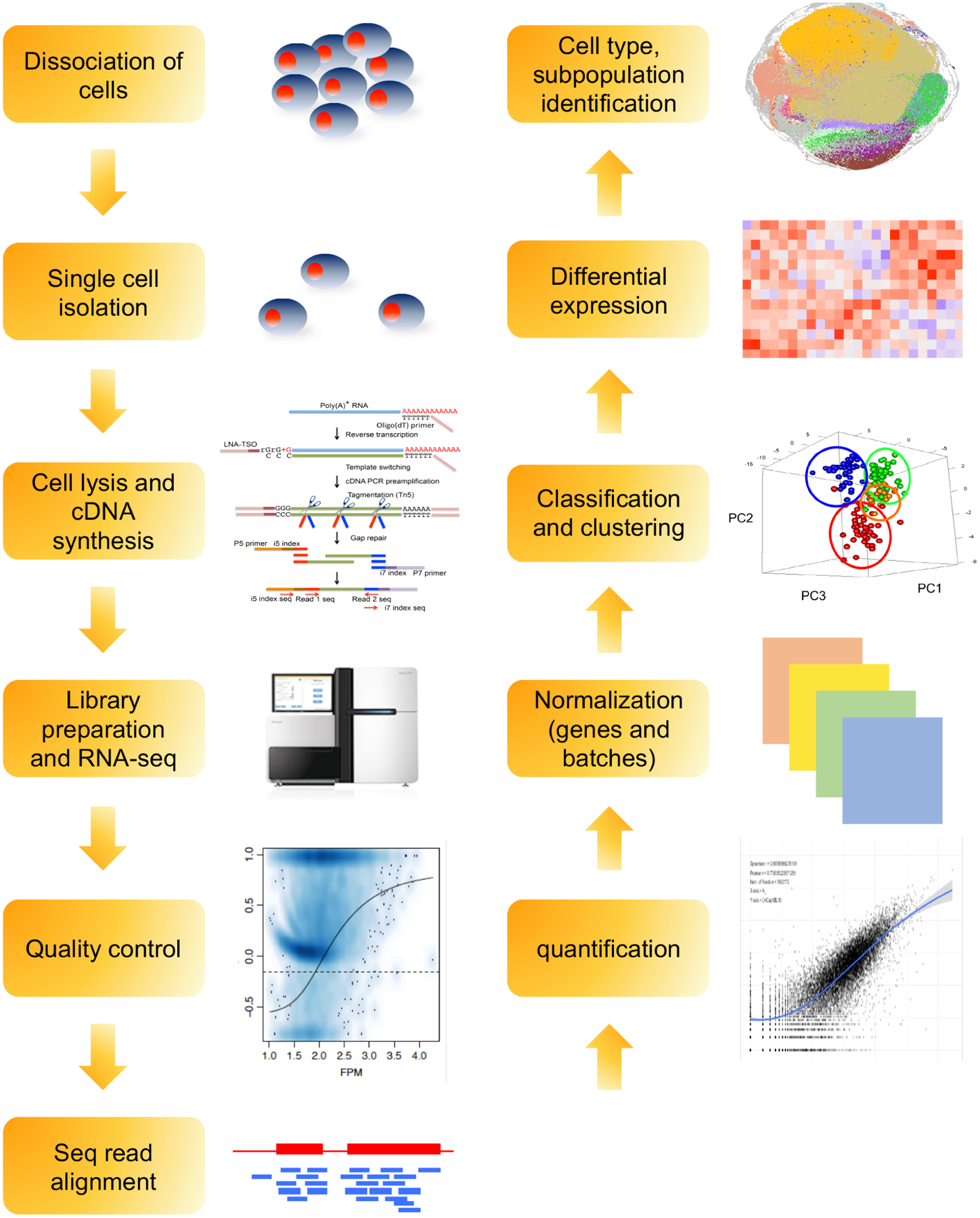
Schematic illustration of scRNA-seq analysis

Synthesized and amplified cDNAs are subsequently subject to library preparation and NGS to generate massive sequencing short reads, as depicted in Figure 2. The current state-of-the-art computational tools and algorithms widely used in bulk RNA-seq analysis can be extended for processing scRNA-seq data. However, the transcriptome at single-cell resolution presents specific analytical challenges, which requires dedicated analytical power and specific packages. Some of the key challenges encountered during single-cell transcriptional data analysis includes greater dimensionality, high level of noise, absence of biological replicates, and data sparsity^[44,75]^. However, major efforts in the development of advanced algorithms and computational strategies as well as adaptations of existing workflows have shown great promise for comprehensive and detailed analysis of scRNA-seq data [Table 2]. Several programming- (R- or Python-based) and web interface-based toolkits have been proposed to facilitate systematic analysis that can be scaled up as per requirements^[75,76]^. Seurat^[77,78]^ and Single Cell ANalysis in PYthon^[79]^ are the two most comprehensive packages that can, respectively, integrate scRNA-seq data with other single-cell data and enable scaling-up to simultaneously analyze millions of cells at the same time. Of note, the core analysis pipelines show higher resemblance with the bulk RNA-seq and can be broadly categorized in the following: (1) quality control; (2) read alignment and generation of counts; (3) removal of confounding factors; and (4) normalization and annotation of cell types and cellular states. The quality of individual single-cell libraries needs to be carefully assessed to abolish the underlying noise as downstream interpretation relies heavily on the preprocessing steps. Generic quality control (QC) metrics including FastQC (http://www.bioinformatics.babraham.ac.uk/projects/fastqc/), High-Throughput Quality Control^[80]^, or Kraken^[81]^ provide insights into overall quality of raw sequence files. Characterization of heterogeneity is one of the primary purposes of performing single-cell analysis, however not all outliers contribute to unique cell populations. Single-cell specific quality evaluation tools such as SinQC^[82]^, SCell^[83]^, and Celloline^[84]^ enable identification of technical artifacts that interfere with gene expression patterns. Mapping sequencing reads to a reference genome or transcriptome allows identification of the specific location from which the transcripts originate and subsequent quantification. Although dedicated mappers for scRNA-seq are not available, existing aligners such as TopHat^[85]^, STAR^[86]^, and Hierarchical Indexing for Spliced Alignment of Transcripts^[87]^ have shown considerable precision and accuracy. Recently, two pseudo-alignment tools, Kallisto^[88]^ and Salmon^[89]^, have been proposed which pseudo-align splicing isoforms to a reference transcriptome and overcome the requirement of significant amount of computational power and time to process the reads. An improved Salmon with Selective Alignment and expanded decoy sequences was introduced recently and significantly reduced false mappings^[90]^. However, careful consideration should be made while implementing pseudo-alignment with scRNA-seq data since the data themselves can have lower depth in the first place and 3’ coverage bias. Another important step in the analysis pipeline is normalization of the expression data, which is particularly important in single-cell analysis as many technical parameters including cell capture efficiency, drop out events, read depth, and coverage bias can induce variation^[91,92]^. Tagging individual RNA molecules using UMIs enables absolute quantification of the transcripts from each cell. In the cases without the use of UMIs, external spike-in RNAs (i.e., ERCCs) can be used as internal controls. Additionally, several single-cell specific normalization approaches including SAMstrt^[93]^, Bayesian Analysis of Single-Cell Sequencing (BASiCS)^[94]^, Gamma Regression Model^[95]^, sctransform^[92]^, Scran^[96]^, SCnorm^[97]^, and Linnorm^[98]^ can be utilized, of which the last three do not require incorporation of additional spike-ins. Eight commonly applied normalization methods (trimmed mean of M-values^[99]^, count-per-million^[100]^, and DESeq2^[101]^, as well as others tailored for scRNA-seq, namely scone, BASiCS, SCnorm, Linnorm, and scran) were subject to benchmarking^[102]^. Data show that scRNA-seq normalization methods outperformed bulk RNA-seq counterparts. However, a bulk RNA-seq normalization method using Differentially Expressed Genes Elimination Strategy is competitive with scRNA-seq normalization methods^[103,104]^. Three scRNA-seq imputation methods, namely K-Nearest Neighbor smoothing (kNN-smoothing)^[105]^, DrImpute^[106]^, and Single-cell Analysis Via Expression Recovery (SAVER)^[107]^, were evaluated for their capacity to tackle the zero-inflation issue either derived from a technical contribution or a normal distribution^[102,108,109]^ [Table 2]. Both kNN-smoothing and DrImpute analysis gave more reliable results compared with SAVER.

Single-cell RNA isolation at different time points or in different laboratories can induce systemic variations and batch effects which may compromise biologically meaningful interpretation of signals^[110,111]^. Batch correcting algorithms such as Mutual Nearest Neighbors Correct^[111]^, Seurat 3^[112]^, Harmony^[113]^, scGen, and scMerge^[114]^, among many others, can compensate the discrepancy. In unsynchronized cells, cell-cycle variation can also mask other important physiological variations, which can be overcome by eliminating cell-cycle factors using packages such as single-cell Latent Variable Model (scLVM)^[115]^ and ccRemover^[116]^. Drop-out, high number of zero counts, sparsity, and multimodality are some of the unique events encountered in single-cell expression analysis which demand more sophisticated algorithms for identifying differentially expressed genes (DEGs)^[117]^.

### Algorithms for scRNA-seq data analysis

Algorithms for scRNA-seq data analysis have been developed recently in different computer languages, such as R program or Python, and few are designed as a website interface or software package [Table 3]. scRNA-seq data are high-dimensional datasets among a great number of cells. Therefore, application of appropriate algorithms is necessary to have better analysis and visualization of scRNA-seq data. After QC and normalization, scRNA-seq data can be processed using diverse algorithms according to variant purposes, such as investigation of DEGs, identification of cell subpopulations, and cell fate trajectories (pseudotime analysis), which are the most common methods to process scRNA-seq data. Visualizations of scRNA-seq data are also diverse. The heatmap is the most common method to present DEGs between groups or within different cell types. Heatmaps are generated by most algorithms for DEGs analysis. T-distributed stochastic neighbor embedding (tSNE), scatter plot, and uniform manifold approximation, and projection (UMAP) are used for visualization of dimension reduction results in cell clustering or cell subpopulations^[118–120]^.

## CELL CLUSTERING AND SUBPOPULATION IDENTIFICATION

Cell clustering and cell type identification are critical features of scRNA-seq and, unlike bulk cell RNA-seq, can reveal heterogeneous cell types using entire transcriptomes from an enormous quantity of cells^[25,44,121]^. Recently, many software algorithms have been developed to achieve cell clustering and cell type identification [Table 3] through unsupervised dimensionality reduction based on principal component analysis (PCA), tSNE, or diffusion maps^[28,122]^. Based on an unsupervised clustering method, such as Seurat or Monocle 3, novel cell types or populations might be revealed with scRNA-seq data^[77,78,123]^. Recently, cell type identification of scRNA-seq data has been exponentially applied to studies in developmental biology, neurology, cancer biology, and immunology and can provide the type, quantity, and gene signature of different cell populations^[124–129]^.

## DIFFERENTIAL EXPRESSION ANALYSIS

Differential expression analysis can reveal significant DEGs to identify novel pathways or biological functions in different cell types or treatments. Identification of DEGs can be performed by comparing gene expression between two predetermined groups or treatments. For example, Horning *et al.*^[130]^ identified a group of cell-cycle genes upregulated in a subpopulation which had an attenuated androgen response using Single Cell Differential Expression (SCDE) algorithm in R program. DEGs can also be identified among different cell types in a tissue or organ based on unsupervised algorithms, such as Seurat or Monocle 3. Wang *et al.*^[131]^ identified single-cell transcriptome profiling of cardiopharyngeal lineages and characterized their cell fate using Seurat package in R program. SCDE^[132]^, PAthway and Gene^[133]^ set OverDispersion Analysis (PAGODA), Model-based Analysis of Single-cell Transcriptomics, Monocle, and SigEMD^[134]^ algorithms and SINgle CEll RNA-seq profiling Analysis^[135]^ workflows have addressed some common challenges to some extent and improved sensitivity in calling DEGs. Since each single cell has the potential to behave as a unique entity, oftentimes the curse of high dimensionality can impose restrictions in clustering and data visualization^[122]^. UMAP, Zero Inflated Factor Analysis, Single-cell Interpretation via Multikernel LeaRning, and scvis belong to recent dimensionality reduction techniques that can address the underlying confounding factors and enable proper visualization of diverse expression patterns over conventional PCA analysis^[120,136,137]^. With the advent of automated advanced tools and packages including SingleR and scMatch, cell-type annotations have significantly improved, leading to the identification of rare events or specific cell populations with the ability to scale-up^[138–140]^. Cell BLAST is a cell type query algorithm for the analysis of new scRNA-seq data. It utilizes a neural network-based generative model to extract low-dimensional cell-to-cell relationships from high-dimensional transcriptomic data and predicts cell types via batch correction with a large-scale curated reference cell type database^[141]^.

## CELL LINEAGE AND CELL FATE RECONSTRUCTION

Following cell type identification, cell fate trajectory is the next step to uncover how different cell types coordinate in many aspects of biology including the developmental process or cancer progression^[142,143]^. Based on transcriptome information, some algorithms provide a pseudotime scale and cell fate branches within all the cells to reveal potential progression or direction of cell types based on cell phenotypic clusters^[123,144]^. Cell fate trajectory analysis provides an opportunity to investigate the dynamic processes of large-scale cells in developmental processes, cellular differentiation, or drug responses^[145,146]^. Several software packages can perform trajectory inference. Monocle 3^[123]^, Diffusion PseudoTime^[144]^, and Single-cell Trajectories Reconstruction, Exploration And Mapping^[145]^ are well-developed algorithms to perform cell fate trajectory prediction but require a mastery of computer programming skills. Tools for Single Cell ANalysis (TSCAN) provides a friendly webpage interface to access and perform cell fate trajectory^[147]^.

Of note, transcriptional dynamics represent an important feature of single-cell analysis which enables the analysis of gene expression in given time series such that the output can generate biological signals inferring potential cellular lineages. Seurat^[77,78]^, Slingshot^[148]^, Monocle 2^[149]^, Waterfall^[150]^, Single-cell Clustering Using Bifurcation Analysis^[151]^, and TSCAN^[147]^ can allow construction of pseudotime trajectory and assessment of expression kinetics that can provide novel insights into cellular differentiation of stem cells as well as oncogenic progression during tumor development. Tian *et al.*^[102]^ evaluated five trajectory analysis methods in a thorough combination of normalization and imputation of four independent scRNA-seq datasets and found that Slingshot and Monocle 2 led to more robust results. Integration of single-cell transcriptome profiling with other single-cell or bulk analysis and spatial measurements can significantly enhance our understanding of molecular basis of cellular heterogeneity^[77]^ and crosstalk among cellular populations in *in-vivo* studies^[152,153]^.

## APPLICATIONS OF SCRNA-SEQ

### Heterogeneity of cell fate determination in embryogenesis

In the last decade, cumulative efforts have been undertaken to explore the uncharted territory of cellular heterogeneity in different species, organs, tissues, developmental stages, and microenvironments. The first attempt was to interrogate the transitional transcription profiles in the formation of pluripotent embryonic stem cells (ESCs) in early embryogenesis^[17]^. The differentiational modulation from the inner cell mass to blastocysts and ESCs was not fully understood based on bulk cell studies, but they provided a good initial model for scRNA-seq analysis^[17,154]^. DEGs showed self-renewal and pluripotency signals with high gene expression variations, particularly for genes with medium expression levels. While epigenetic repressor expression was increased, a suppressive transcription became apparent during the development. A group of miRNAs targeting early differentiation genes and pluripotency genes plays a role in transcriptional alterations. Meanwhile, many spliced forms were discovered for the first time. The same approach was also applied to profile the transcriptional dynamics of earlier embryonic stages, preimplantation human embryos, using 124 cells from oocyte to blastocyst stage^[155]^. Previous bulk studies have shown that the expressions of ~1900 genes were mainly transcriptionally suppressed during the stages. In this study, about 2,495 and 2,675 genes were significantly up- and downregulated between the four- and eight-cell stages. Splicing isoforms of 4,822 transcripts were enriched in different stages and 20% of transcripts displayed more than two splicing variants. FOXP1 with exon 18b transcripts, ESC-specific splicing species, are 25-fold more abundant than those with exon 18a in undifferentiated ESCs. From the 90 single embryonic cells analyzed, 64% of the total known human lncRNAs (28,640) were found to be expressed. Another study using Smart-seq method with deep sequencing analyzed the cell fate determination between two- and four-cell embryos and later stage blastomeres^[156]^. However, this scRNA-seq study discovered that dozens of protein-encoding genes, including Gadd45a, showed significant differential bimodal expression between blastomeres at two- and four-cell embryonic stages. Differential monoallelic expression in 24% genes was clearly observed to be independently regulated in early embryonic development using scRNA-seq^[157]^. In later embryonic developmental stages within 5–7 days, X-chromosome dose compensation was found in single-cell transcriptomes of 1,529 individual cells from 88 human preimplantation embryos^[158]^. The cell lineage expression patterns were concurrent as an intermediate status before the establishment of the trophectoderm, epiblast, and primitive endoderm lineages that are contemporary with blastocyst formation. Linnarson’s group studied the differential expression between mouse embryonic stem cells and fibroblasts with a high throughput scRNA-seq method^[18]^. Nematode embryogenesis between two- and eight-cell stage was dissected with CEL-seq with RNA linear amplification by scoring single-cell transcriptomes^[54]^. Seventeen genes had significant two-fold mean difference between AB and P1 cells. EMS had more genes expressed compared to P2 cells, while P3 had fewer new genes expressed than C lineage. Taken together, the single-cell transcriptome data map the cell fates in early embryonic differentiation and ESC pluripotency establishment.

### Heterogeneity in complex differentiated tissues and systems

With multiplexing and high throughputs improvements, scRNA-seq has served as a molecular scalpel directed at the heterogeneity of cells in much more complex tissues, systems, and organisms. The immune system is a complex of bone-marrow-derived differentiated cells. More and more scRNA-seq technologies are adopted for exploring transcriptomes and functional relevance in this biological system^[159]^. Dendric cells (DCs) are a group of highly heterogeneous antigen-presenting cells and important for pathogen recognition and immune defense^[160]^. The bulk RNA-seq of marker-sorted subpopulations did not sufficiently capture their complex functions and led to great controversy^[161]^. An unbiased global transcriptomic mapping of 18 bone-marrow-derived DCs exposed to lipopolysacharrides (LPS)^[162]^ using Smart-seq revealed hundreds of genes expressed in high variability and unique bimodal profiles that were similarly observed during early mammalian embryogenesis^[13,156]^. Among them, 137 genes are anti-virus genes. The spleen is the largest lymphatic organ in the human body. The heterogeneity of 1,536 splenic cells was explored using massively parallel MART-seq with low-depth RNA sampling^[61]^. From them, the method coupled with a probabilistic mixture model demonstrated sensitive cell classification for distinct identification of B cells, natural killer cells, macrophages, monocytes, and plasmacytoid DCs. In DCs, four subpopulations were found either significantly linked or supported by internal combinatorial marker gene expressions. After exposure to LPS, 1,536 spleen cells’ scRNA-seq displayed the heterogeneity in DCs with enriched CD11c expression and their response to LPS. In the adaptive immune system, differentiation of naïve T cells into T helper 2 (Th2) cells is a feedback loop to restrain immune overreaction^[163]^. From 91 single Th2 cells acquired post infection of naïve T cells, scRNA-seq revealed unique subpopulations with transcriptional profiles and changes in transcription factors, cytokines, surface receptors, and other pathways^[115,164]^.

Mining efforts on heterogeneity of other tissues are ongoing in muscle, lung, intestine, testis, pancreas, and the nervous system. The sci-RNA-seq was applied to profile nearly 50,000 cells from nematodes (*Caenorhabditis elegans*) with more than 50-fold somatic cellular coverage at the L2 larval stage^[67]^. From the data, consensus expression profiles for 27 cell types were defined and rare neuronal cell types with one or two cells were sensitively recovered. The global view of regulatory networks for human skeletal muscle myoblast differentiation has been masked by the low resolution of bulk genomic data^[149]^. ScRNA-seq coupled with a nonlinear MONOCLE pseudotime trajectory prediction model discovered dynamic expression in 1,061 genes that clustered in gene regulatory groups responsible for activation and suppression at three time points after differentiation initiation. During Embryonic Days 16.5–18.5, murine lung cell lineages at respiratory airway tips are developed from columnar epithelial progenitor cells into flat alveolar type 1 (AT1) or cuboidal type 2 (AT2) cells for gas exchange or surfactant secretion, respectively^[165]^. A few markers have been identified for four cell types but the global transcriptomic dynamics during the transition is unknown^[166]^. Microfluidic scRNA-seq of 196 cells have delineated transcriptional signatures for an intermediate bipotential progenitor cells that precede AT1 and AT2 cells, in addition to Clara and ciliated cells^[167]^. CEL-seq of 238 randomly selected cells from intestinal organoids composed of major intestinal cell lineages brought a better understanding of diversity in intestinal differentiation^[168]^. Hierarchical clustering of gene expression correlation and rare cell identification method identified the major intestinal cell lineages and 10 clusters as novel diverse subtypes of cells. Spermatogenesis in testes is a complicated and highly orchestrated process including the differentiation of diploid spermatogonia into haploid sperm^[169]^. The whole picture of spermatogenesis is still far from complete. Two research groups have run scRNA-seq on thousands of dissociated cells from testis samples using Drop-seq and STAR^[170,171]^. A conserved continuous temporal trajectory of transcriptional dynamics was consistent in both murine and monkey reproductive models. Novel subpopulations were identified in several time points of differentiation and displayed unique transcriptional regulators and signatures. Based on CEL-seq2 data of pancreatic islet cells from four deceased patients, cell clusters by t-distributed Stochastic Neighbor Embedding (t-SNE) analysis showed the classical pancreatic cell types with marker genes and additional novel markers that have not been reported previously^[172]^.

The central nervous system is composed of large amounts of neuronal and glial cells with numerous types, and the classical methods to identify them with some molecular markers were limited and not definitive^[173]^. Single-cell transcripts of ~3000 cells from mouse somatosensory S1 cortex and hippocampus *Cornu Ammonis* (CA) were analyzed by STRT/C1^[174]^. Cell type classification identified nine major classes and 47 molecularly distinct subclasses. scRNA-seq of 30,000 nuclei from mouse and human archived brain tissues from hippocampus and prefrontal cortex was carried out by DroNc-seq^[66]^. With fewer genes detected, cell clustering analysis still identified novel cell types along with well-known cell types.

In other independent studies, there were more than 100 subclasses of cells found in mouse brain and spinal cord^[68,175]^. Ribosomes And Intact Single Nucleus (RAISIN) RNA-seq and MIning RAre Cells sequencingMIRACL-seq processed transcriptomes of thousands of neurons in mouse and human enteric nervous system for species-specific transcription signatures and dozens of neuronal subtypes^[176]^. From 44,808 mouse retinal cells, 39 transcriptionally distinct cell populations were identified, creating an atlas of gene expression for the classification of retinal cells and novel rare subtypes^[33]^.

### Heterogeneity in cancers

The transcriptomic heterogeneity of tumors evolves temporospatially during tumor progression with genetic, epigenetic, and tumor immune microenvironmental fluctuations^[5,7,177]^. ScRNA-seq is a powerful tool to address the tumoral heterogeneity, particularly for rare cells and previously unrecognizable subpopulations^[128]^. Smart-seq was applied to stratify heterogenous cell subpopulations in 672 cells from five glioblastoma tumors^[14]^. Despite apparent cell-to-cell variability, unbiased cell hierarchical clustering showed four meta-signatures comprised of cell-cycle, hypoxia, complement/immune response, and oligodendritic function. Gene expression profiling of 4,347 cells from six Isocitrate dehydrogenase 1(IDH1) or IDH2 mutant human oligodendrogliomas displayed distinct expression signatures^[178]^. With bulk exome sequencing and copy number variation estimation, a hierarchical cell lineage map with variant stem/progenitor cell components was delineated in each tumor. Noncanonical WNT activation signaling was noted in retrospective analysis of 77 circulating tumor cells from 13 prostate cancer (PCa) patients following tumor progression compared with stable counterparts undergoing androgen deprivation therapy^[179]^. This study indicated a potential novel therapeutic target and predictive biomarker for PCa. From multicellular ecosystem of metastatic melanoma, 4,645 single cells isolated from 19 patients were subject to analysis for profiling malignant, immune, stromal, and endothelial cells^[180]^. The principle component analysis of scRNA-seq data showed that the transcriptomic expression could discern malignant cells from tumor and nonmalignant cells (immune cells, stromal cells, endothelial cells, and fibroblasts) independent of biopsy sites. The transcriptional signatures for malignant cells consist of a core set of cell-cycle genes and a set of immediate early-activation transcription factors that displayed spatial difference. Meanwhile, a drug-resistant subpopulation with high AXL or MITF signals was present in treatment-naive tumors. Treatment-naïve tumors are usually sensitive to initial therapy and generally respond to first-line therapy. However, most advanced tumors acquire drug resistance and lead to poor survival outcomes. Androgen deprivation therapy^[8]^ is effective for the majority of PCa but biochemical recurrence occurs in 30% of patients subject to treatment, and there is a limited understanding of the underlying mechanisms. From 144 cells treated or untreated with androgen, subpopulations of heterogeneous LNCaP cells were revealed and exhibited high levels of ten cell-cycle-related genes using Smart-seq2 analysis^[130]^. The subpopulations of cells showed cancer stemness phenotype and became resistant to cell-cycle targeting agents. ScRNA-seq and imaging found transcriptional variation and a pre-adapted subpopulation that exhibited resistance to endocrine therapy^[181]^. ScRNA-seq identified a stem-like subpopulation of PCa cells from monolayer and organoid culture^[182]^.

Smart-seq2 was deployed to sequence single cells derived from treatment naïve, residual disease, and progressive disease following tyrosine kinase inhibitor (TKI)-based therapies in tumor derived from non-small cell lung cancer patients for mapping transcriptional alterations unique to drug-sensitive and drug-resistant tumor cell populations^[183]^. The scRNA-seq data of 23,261 cells from 49 samples show high-power resolution of high cellular heterogeneity and that residual disease tumors have fewer proliferative markers and increased alveolar cell markers. In TKI-resistant tumors, the upregulated genes were related to oncogenesis and inflammation. Moreover, progressive disease had increased infiltration of immune cells, predominant MF2 macrophages, and suppressive T cells in tumor microenvironments.

Melanoma-associated immune and stromal cells were isolated and analyzed by Smart-seq2 at three time points during tumor development^[184]^. The three temporal subpopulations of stromal cells displayed unique functional signatures. The lymphocytes from lymph nodes underwent activation and clonal expansion in tumors. To map the heterogeneity in the immune cells within hepatocellular carcinoma tumors, scRNA-seq methods were used to study CD45^+^ cells isolated from tumors and four immune-relevant sites of 16 treatment-naïve liver cancer patients^[129]^. it was found that LAMP3^+^ dendric cells contain unique transcriptional features affecting other immune cell types and show the ability to migrate to lymph nodes. Exhibiting distinct transcriptional states, tumor-associated macrophages were associated with poor prognosis^[185]^. The inflammatory roles of SLC40A1 and GPNMB were clearly demonstrated in these cells.

## CONCLUSION

Cell heterogeneity has been more appreciated under the light of a new paradigm due to the advances of scRNA-seq and other single-cell analysis technologies. Since its induction, scRNA-seq has been well received and undergone fast-paced technical advances in uniform cDNA amplification, length coverage, rare copy detection, multiplexing, high throughput, processing of metadata, DEG calling, cell clustering, subpopulation identification, and cell fate trajectory predictions. Along with the new technology progress with higher sensitivity and accuracy, our understanding about the extent of cellular heterogeneity has been swiftly updated and repeatedly brought to another level. The discovery of new cell subpopulations and rare cell types with transcriptomic signatures posit new mechanisms for cell functions and defects that lead to novel biomedical applications and rising therapeutic venues.

## Figures and Tables

**Table 1.** cDNA synthesis and amplification techniques for scRNAseq

Methods	coverage	UMI	Strand specific	cDNA synthesis	Detected genes	References
Tang’s	Nearly full-length	No	No	poly(T) primer	13K	Tang *et al.*^[43]^, 2009
STRT-seq and STRT/C1	3’and 5’-only	Yes	Yes	tailed oligo-dT primer; a barcoded r(G)_3_ helper oligo primer	~2–4K	Islam *et al.*^[18,48]^, 2011, 2014
Smart-seq	Full-length	No	No	tailed oligo(dT) priming using the	~8K	Ramskold *et al.*^[49]^, 2012
				CDS primer		
CEL-seq (CEL-seq2)	3’-only	Yes	Yes	8bp-barcoded poly(T) primer	~5K	Hashimshony *et al.*^[54,55]^, 2012; 2016
Smart-seq2	Full-length	No	No	tailed oligo(dT) priming using the CDS primer	~10K	Picelli *et al.*^[50,51]^, 2013; 2014
Quartz-Seq	Full-length	No	No	poly(T) primer	5.8–6.3K	Sasagawa *et al.*^[60]^, 2013
DP-seq	3’-only	No	No	hexamer	11K transcripts	Bhargava *et al.*^[69]^, 2013
SCRB-seq	3’ only	Yes	Yes	cell-barcoded UMI-Poly(T) primer	3k transcripts	Soumillon *et al.*^[56]^, 2014
MARS-seq	3’-only	Yes	Yes	barcoded Poly(T) primer	~200–1500 transcripts	Jaitin *et al.*^[61]^, 2014
Drop-seq	3’-only	Yes	Yes	bead-based barcoded UMI-poly(T) primer	6–7K genes	Macosko *et al.*^[33]^, 2015
InDrop	3’-only	Yes	Yes	hydrogel sphere encapped cell barcoded UMI-poly(T)	29KUMIFM	Klein *et al.*^[34]^, 2015
SUPeR-seq	Full-length	No	No	Random (AnchorX-T15N6) primers	~10K	Fan *et al.*^[65,186]^,2O15
CytoSeq	3’-only	Yes	Yes	Illumina universal PCR primer & cell UMI-Poly(T)	~100	Fan *et al.*^[65]^, 2015
SC3-seq	3’ only	No	No	V1(dT)24	4–6K	Nakamura *et al.*^[70]^, 2015
MATQ-seq	Full-length	Yes	Yes	GATdT primers; MALBAC primers	~14K	Sheng *et al.*^[57]^, 2017
Chromium	3’-only	Yes	Yes	Gel bead based 14x GEM index-lOx barcoded-poly(T) primer	~500	Zheng *et al.*^[63]^, 2017
SPLiT-seq	3’-only	Yes	Yes	random hexamer and anchored poly(dT)15 barcoded RT primers	4.5.−5.5K	Rosenberg *et al.*^[68]^, 2018
sci-RNA-seq	3’-only	Yes	Yes	10bp barcoded-8bp UMI- Poly (T)30 primer	4–5.5K	Cao *et al.*^[67]^, 2017
Seq-Well	3’-only	Yes	Yes	bead-based 12bp barcoded 8bp UMI- Poly(T)30 primer	6–7 K	Gierahn *et al.*^[64]^, 2017
DroNC-seq	3’-only	Yes	Yes	bead-based barcoded UMI-poly(T) primer	1.7–3.3K	Habib *et al.*^[66]^, 2017
Quartz-Seq2	3’-only	Yes	Yes	cell-barcoded UMI-poly(T) primer (v3.1:73-mer)	8K	Sasagawa *et al.*^[187]^, 2018

STRT-seq: single-cell tagged reverse transcription sequencing; CEL-seq: cell expression by Linear amplification and sequencing; DP-seq: designed primer-based RNA-sequencing; SCRB-seq: single cell RNA barcoding and sequencing; MARS-seq: MAssively parallel RNA single-cell sequencing; MATQ-seq: Multiple annealing and dC-tailing-based quantitative single-cell RNA-seq; SPLiT-seq: split-pool ligation-based transcriptome sequencing

**Table 2.** NGS data analysis tools and software for scRNA-seq

Category	Tools	Software	References
Quality control	MultiQC	http://multiqc.info	Ewels *et al.*^[188]^,2016
	SinQC	http://www.morgridge.net/SinQC.html	Jiang *et al*.^[82]^, 2016
	SCell	https://github.com/diazlab/SCell	Diaz *et al.*^[83]^, 2016
	Celloline	https://github.com/Teichlab/celloline	Llicic *et al.*^[84]^,2016
	Kraken	http://ccb.jhu.edu/software/kraken/	Wood and Salzberg^[81]^ 2014
	HTQC	https://sourceforge.net/projects/htqc/	Yang *et al.*^[80]^, 2013
	FastQC	https://www.bioinformatics.babraham.ac.uk/projects/fastqc/	2010
Alignment	Kallisto	https://github.com/pachterlab/kallisto	Bray *et al.*^[88]^, 2016
	HISAT	https://github.com/infphilo/hisat	Kim *et al.*^[87]^, 2015
	TopHat2	https://github.com/infphilo/tophat	Kim *et al.*^[189]^, 2013
	STAR	https://code.google.com/archive/p/rna-star/	Dobin *et al.*^[86]^, 2013
	GSNAP	https://bioinformaticshome.com/tools/rna-seq/descriptions/GSNAP.html	Wu *et al.*^[190]^, 2010
	MapSplice	http://www.netlab.uky.edu/p/bioinfo/MapSplice	Wang *et al.*^[191]^, 2010
Quantification	StringTie	http://ccb.jhu.edu/software/stringtie/	Pertea *et al.*^[192]^, 2015
	HTSeq	https://htseq.readthedocs.io/en/master/	Anders *et al*.^[193]^, 2014
	FeatureCounts	http://subread.sourceforge.net	Liao *et al.*^[194]^, 2013
	RSEM	http://deweylab.github.io/RSEM/	Li and Dewey^[195]^, 2011
	Cufflinks	http://cole-trapnell-lab.github.io/cufflinks/	Trapnell *et al.*^[196]^, 2010
Normalization	sctransform	https://github.com/ChristophH/sctransform	Hafemeister and Satija^[92]^, 2019
	SCnorm	https://github.com/rhondabacher/SCnorm	Batcher *et al*.^[97]^, 2017
	Linnorm	http://www.jjwanglab.org/linnorm	Yip *et al.*^[98]^, 2017
	SCran	https://rdrr.io/bioc/scran/	Lun *et al.*^[96]^, 2016
	BASiCS	https://github.com/catavallejos/BASiCS	Vallejos *et al.*^[94]^, 2015
	GRM	http://wanglab.ucsd.edu/star/GRM/	Ding *et al.*^[95]^, 2015
	SAMstrt	https://github.com/shka/R-SAMstrt	Katayama *et al.*^[93]^, 2013
Analysis pipeline	Seurat	https://github.com/satijalab/seurat	Butler *et al*.^[77]^, 2018
	SCANPY	https://github.com/theislab/Scanpy	Wolf *et al.*^[79]^, 2018
	Scater	https://rdrr.io/github/davismcc/scater/	McCarthy *et al*.^[197]^, 2017
	Granatum	https://github.com/lanagarmire/Granatum	Zhu *et al.*^[198]^, 2017
	ASAP	https://github.com/DeplanckeLab/ASAP	Gardeux *et al.*^[199]^, 2017
	SCran	https://rdrr.io/bioc/scran/	Lun *et al.*^[96]^, 2016
	SINCERA	https://research.cchmc.org/pbge/sincera.html	Guo *et al.*^[135]^, 2015
Batch correction	Seurat 3	https://github.com/satijalab/seurat	Stuart *et al.*^[112]^, 2019
	Harmony	https://github.com/immunogenomics/harmony	Korsunsky *et al.*^[113]^, 2019
	scGEN	https://github.com/theislab/scgen	Lotfollahi *et al.*^[200]^, 2019
	scMerge	https://sydneybiox.github.io/scMerge/	Lin *et al.*^[114]^, 2019
	MNN Correct	https://github.com/MarioniLab/MNN2017/	Haghverdi *et al.*^[111]^, 2018
Alternative splicing	Expedition	https://github.com/YeoLab/Expedition	Song *et al.*^[201]^, 2017
	BRIE	https://github.com/huangyh09/brie	Huang and Sanguinetti^[202]^, 2017
	Census	https://github.com/cole-trapnell-lab/monocle-release	Qiu *et al.*^[203]^, 2017
	SingleSplice	https://github.com/jw156605/SingleSplice	Welch *et al*.^[204]^, 2016
Other	ccRemover	https://cran.r-project.org/web/packages/ccRemover/index.html	Barron and Li^[116]^, 2016
cofounding	scLVM	https://github.com/PMBio/scLVM	Buettner *et al.*^[115]^, 2015
factor removal	COMBAT	https://github.com/Jfortin1/ComBatHarmonization	Johnson *et al*.^[205]^, 2007

HTQC: high-throughput quality control; HISAT: hierarchical indexing for spliced alignment of transcripts; BASiCS: bayesian analysis of single-cell sequencing; GRM: gamma regression model; SCANPY: single cell analysis in python; SINCERA: SINgle cell RNA-seq profiling analysis; MNN: mutual nearest neighbors; scLVM: single-cell latent variable mode

**Table 3.** Software/packages for single-cell RNA-seq analysis: differential expression, subpopulation identification, clustering, and peudotime projection

Software/package	Differential expression	Clustering cell type	Cell fate trajectories	Language	Programing skill	Reference
PAGODA	Yes	Yes	No	R	+++	[133]
SCDE	Yes	No	No	R	+++	[132]
Seurat	Yes	Yes	No	R	+++	[77,112]
SCENIC	Yes	Yes	No	R or Python	+++	[132]
Destiny	No	Yes	Yes	R	++	[206]
TSCAN	Yes	no	Yes	R or website interface	+	[147]
Monocle 3	Yes	Yes	Yes	R	+++	[123]
Waterfall	Yes	no	Yes	R	+++	[150]
Wishbone	No	No	Yes	Python	+++	[29]
GrandPrix	No	Yes	Yes	Python	+++	[207]
DPT	No	No	Yes	R or Python	+++	[144]
SCUBA	No	Yes	Yes	MATLAB	+	[151]
STREAM	No	Yes	Yes	Python	+++	[145]
Slingshot	Yes	Yes	Yes	R	+++	[148]
CellRouter	Yes	Yes	Yes	R	+++	[208]

PAGODA: pathway and gene set overdispersion analysis; SCDE: single cell differential expression; TSCAN: tools for single cell analysis; DPT: diffusion pseudotime; SCUBA: single-cell clustering using bifurcation analysis; STREAM: single-cell trajectories reconstruction, exploration and mapping
